# Foveal neural adaptation to optically induced contrast reduction

**DOI:** 10.1167/jov.24.9.13

**Published:** 2024-09-17

**Authors:** Antonia Roth, Katharina Breher, Niklas Domdei, Siegfried Wahl

**Affiliations:** 1 Institute for Ophthalmic Research, University of Tübingen, Tübingen, Germany; 2 Carl Zeiss Vision International GmbH, Aalen, Germany

**Keywords:** contrast adaptation, neural processing, contrast sensitivity, scattering, contrast reduction

## Abstract

Contrast processing is suggested to interact with eye growth and myopia development. A novel contrast-reducing myopia control lens design decreases image contrast and was shown to slow myopia progression. Limited insights exist regarding neural visual processing following adaptation to image contrast reduction. This study investigated foveal neural contrast sensitivity in 29 young adults following a 30-minute adaptation to scattering using a Bangerter occlusion foil 0.8, +0.5-diopter defocus, and a clear lens control condition. Neural contrast sensitivity at its peak sensitivity of 6 cycles per degree was assessed before and after adaptation to the lens conditions, employing a unique interferometric system. Pre-adaptation measurements were averaged from six replicates and post-adaptation measurements by the first and last three of six replicates. The change in neural contrast sensitivity was largest for scattering across the first and last three post-adaptation measurements (+0.05 ± 0.01 logCS and +0.04 ± 0.01 logCS, respectively) compared with control and defocus (all +0.03 ± 0.01 logCS). For scattering, the observed increase of neural contrast sensitivity within the first three measurements differed significantly from the pre-adaptation baseline (*p* = 0.04) and was significantly higher compared with the control condition (*p* = 0.04). The sensitivity increases in the control and defocus conditions were not significant (all *p* > 0.05). As the adaptation effect diminished, no significant differences were found from baseline or between the conditions in the last three measurements (all *p* > 0.05). When post-adaptation neural contrast sensitivities were clustered into 25-second sequences, a significant effect was observed between the conditions, with only a significant relevant effect between control and scattering at 25 seconds (*p* = 0.04) and no further significant effects (all *p* > 0.05). The alteration in neural contrast sensitivity at peak sensitivity was most pronounced following adaptation to the scattering condition compared with defocus and control, suggesting that induced scattering might be considered for myopia control.

## Introduction

Myopia is one of the most common ocular disorders (Holden et al., 2016) based on the imbalance of the ocular axial length and the refractive power of the cornea and lens (Atchison, Schmid, & Pritchard, 2006). Greater eye growth corresponds to an elevated risk of developing various eye diseases (e.g., choroidal thinning, retinal detachment, glaucoma, myopic maculopathy), ultimately resulting in visual impairment (Ohno-Matsui et al., 2021; Vera-Diaz, Bex, Ferreira, & Kosovicheva, 2018).

A need to control myopia progression exists. Thus, myopia control strategies aim to minimize abnormal axial elongation of the eye. Among environmental and pharmacological strategies, various optical strategies exist (Kaiti, Shyangbo, Sharma, & Dahal, 2021). The latter can be divided into contact lenses and spectacle lenses, where spectacles present a non-invasive myopia treatment solution. Current spectacle lenses are designed based on three hypotheses: (a) a mutation in either the L- or M-cone opsin results in an abnormally high contrast signal, which in turn interferes with the eye growth control loop (Hagen et al., 2019; Rappon et al., 2022a); (b) imposed peripheral myopic defocus slows axial elongation (Berntsen, Barr, Mutti, & Zadnik, 2013; Walline et al., 2020); and (c) decreasing the lag of accommodation minimizes hyperopic defocus and slows myopia progression (Logan et al., 2021; Seidemann & Schaeffel, 2003). These three hypotheses collectively suggesting that altering retinal image quality induced by myopia control lenses might reset the growth control loop (Rappon, Neitz, & Neitz, 2020; Rappon, Neitz, Neitz, & Chalberg, 2022b; Rappon, Neitz, Neitz, Chung, & Chalberg, 2022c).

A recent study investigated parameters such as sharpness and contrast via optical characterization of scattering lenses (diffusion optics technology [DOT]) and defocus lenses (defocus incorporated multiple segments [DIMSs]). For the scattering lens, a decrease in peripheral contrast and sharpness was found, related to hypothesis (a) (Arias, Ohlendorf, Artal, & Wahl, 2023; Pérez, Archer, & Artal, 2010). For the defocus lens, an increase in peripheral contrast and sharpness was found, referred to as hypothesis (b) (Arias et al., 2023). Hypothesis (c) is applied centrally and improves the foveal but not peripheral image quality, which is achieved via progressive addition lenses. This suggests distinct underlying mechanisms for each lens strategy.

Thus, current myopia research is focused on understanding the central and peripheral retinal processes involving scattering and defocus, whereby myopia control lenses are designed to influence the retinal periphery. A clinical trial investigating induced scattering through DOT lenses (SightGlass Vision, Inc., 2023) has shown promising results, reporting reduced progression in myopic refractive error and axial elongation (Rappon, Neitz, & Neitz, 2020; Rappon et al., 2022c). In contrast, animal studies found form-deprivation myopia after adaptation to scatter lenses or diffusers (Bowrey, Metse, Leotta, Zeng, & McFadden, 2015; Fitzgerald, Wildsoet, & Reiner, 2002; Flitcroft, Harb, & Wildsoet, 2019; Smith & Hung, 2000; Smith et al., 2007; Tran, Chiu, Tian, & Wildsoet, 2008). Investigating DIMS lenses, a human clinical trial also exhibited a reduction in myopia progression (Lam et al., 2020). In general, previous literature has shown that full-field-of-view exposure to positive lenses slows myopia progression (Berntsen et al., 2013; Logan et al., 2021; Seidemann & Schaeffel, 2003; Walline et al., 2020), as the peripheral image is focused on the retina, whereas negative lenses, focusing the image behind the retina, promote myopia progression (Leube, Ohlendorf, & Wahl, 2016; Ohlendorf & Schaeffel, 2009; Thibos, Bradley, Liu, & López-Gil, 2013).

The visual system is, among others, able to adapt to alterations in contrast and blur, which has been the subject of recent extensive myopia research (Sankaridurg et al., 2023; Vera-Diaz et al., 2018). Contrast adaptation, defined by an altered contrast sensitivity, occurs due to the recalibration of spatial frequency channels and therefore changes the sensitivity of the visual system (Blakemore & Campbell, 1969b). Insufficient and contradictory information is available about short-term adaptation processes to scattering in humans. The literature to date shows that short-term adaptation to scattering leads, on the one hand, to an improvement in visual performance (Villa-Carpes, Bueno, & Fernández, 2021), whereas, on the other hand, eye lengthening (Teoh, Collins, Read, & Pieterse, 2021) and a decrease in contrast sensitivity (Breher, Neumann, Kurth, Schaeffel, & Wahl, 2023; Neumann et al., 2022) have been reported. Furthermore, the underlying mechanisms are not yet clear and require further investigation.

Contrast adaptation to myopic defocus is associated with thickening of the choroid and thus controlling myopia by slowing down the lengthening of the eye (Sankaridurg et al., 2023). In this context, short-term adaptation is thought to provide a hint regarding the success of the applied myopia control strategy (Ghosh, Zheleznyak, Barbot, Jung, & Yoon, 2017; Ohlendorf & Schaeffel, 2009). The increase in sensitivity of the contrast channels due to the adaptation process plays a crucial role in the signal cascade in regulating eye growth (Diether, Gekeler, & Schaeffel, 2001; Mon-Williams, Tresilian, Strang, Kochhar, & Wann, 1998).

However, studies investigating contrast sensitivity are limited by the influence of the optical properties of the eye (Ghosh et al., 2017), altering the individually perceived stimulus contrast. This limitation can be circumvented via laser interferometry, which allows for an aberration-free stimulation by bypassing the optics of the eye and therefore assessment of the neural contrast sensitivity (Campbell & Green, 1965; Suchkov, Kurian, Schwarz, Leube, & Wahl, 2021).

The purpose of this study was to investigate how neural contrast sensitivity adapts to reduced retinal image contrast caused by scattering or defocus at the sensitivity peak (Blakemore & Campbell, 1969a; Michael, Guevara, de la Paz, Alvarez de Toledo, & Barraquer, 2011). The study aims to identify changes in neural contrast sensitivity following exposure to these conditions, which are relevant strategies in myopia control. To elaborate the persistence of potential effects, these changes are analyzed over time. The results can provide insights into neural processing after adaptation to the lens conditions. In addition, it allows the evaluation of potential differences in neural processing between the scattering and defocus and whether the use of such strategies could be beneficial in controlling myopia progression.

## Material and methods

### Study participants

This prospective study was conducted at the Institute for Ophthalmic Research at the University of Tübingen. The study protocol followed the tenets of the Declaration of Helsinki and data protection regulations. Approval was obtained from the ethics committee of the Faculty of Medicine of the University of Tübingen. Before the measurements, participants signed a written informed consent form after the content and possible consequences of the study had been explained. Inclusion criteria were self-reported systemic and ocular health, corrected visual acuity of ≤0.1 logMAR, and age between 18 and 40 years. Participants were excluded who had astigmatism > 2 diopters (D), anisometropia of >1 D, or a history of orthokeratology wear or refractive surgery. Additional measurements were conducted to confirm inclusion criteria. Here, screening for corneal and retinal abnormalities was performed using wavefront aberrometry (ZEISS i.Profiler^plus^; Carl Zeiss Vision, Aalen, Germany), biometry (ZEISS IOLMaster 700; Carl Zeiss Meditec, Jena, Germany), and optical coherence tomography (ZEISS PLEX Elite 9000; Carl Zeiss Meditec, Dublin, USA). Subjective refraction was carried out using ZEISS VISUSCREEN 500 and ZEISS VISUPHOR 500 (Carl Zeiss Vision) to fully correct the subjective spherical and astigmatic refractive errors with a trial frame (Oculus B5; Oculus, Wetzlar, Germany) and trial lenses (Oculus BK 1/T; Oculus) in a vertex distance of 12 mm.

### Neural contrast adaptation assessment

**Figure 1.**
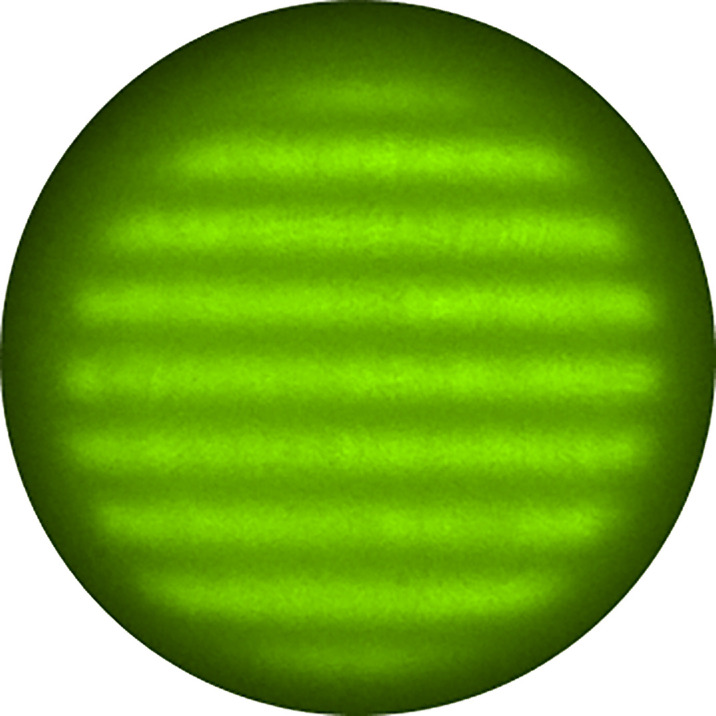
The high-contrast 1.5° stimulus (550 ± 5 nm, 300 Td) containing horizontal fringes at a spatial frequency of 6 cpd.

Neural contrast sensitivity was psychophysically assessed with an interferometric system that enables projecting aberration-free gratings directly on the retina (Campbell & Green, 1965; Williams, 1985). The setup is described in detail elsewhere (Breher, Gottschalk, Domdei, & Wahl, 2022; Suchkov et al., 2021). In brief, the key element of the system, a spatial light modulator, splits the light of a coherent light source via phase mask into two laterally separated wavefronts. These two beams are focused on the pupil plane of the eye, diverge behind, and form interference fringes on the retina. The advantage of this test procedure is that optical aberrations of the eye can be neglected due to the Maxwellian view configuration, and tonic accommodation does not need to be controlled. In the study procedure, a 1.5° narrowband stimulus grating with a wavelength of 550 ± 5 nm (300 Td) was used, containing horizontal fringes with a spatial frequency of 6 cycles per degree (cpd) (Figure 1). To avoid adaptation to the stimulus during the adjustment procedure, the stimulus was flashed with a flickering frequency of 1 Hz. The psychophysical testing procedure was based on the method of adjustment programmed in MATLAB R2022a (MathWorks, Natick, MA). Each trial of the neural contrast sensitivity test began with a subthreshold contrast setting of −2.5 log. The contrast threshold was determined by the participants, who adjusted the contrast so that the stimulus was just visible, from subthreshold contrast (not seen) to threshold contrast (barely seen). Two possible contrast adjustment step sizes were implemented: 0.05 log and 0.20 log. This study included six stimulus presentations per measurement run. An individual bite bar was prepared to align the participant's eye with the pupil plane of the system. An on-axis pupil camera (DMK 27AUP031; The Imaging Source Europe, Bremen, Germany) was used to monitor the ideal position of the participant's eye, which, if necessary, was corrected to ensure a stable stimulus formation on the central retina during testing.

### Adaptation conditions

**Figure 2.**
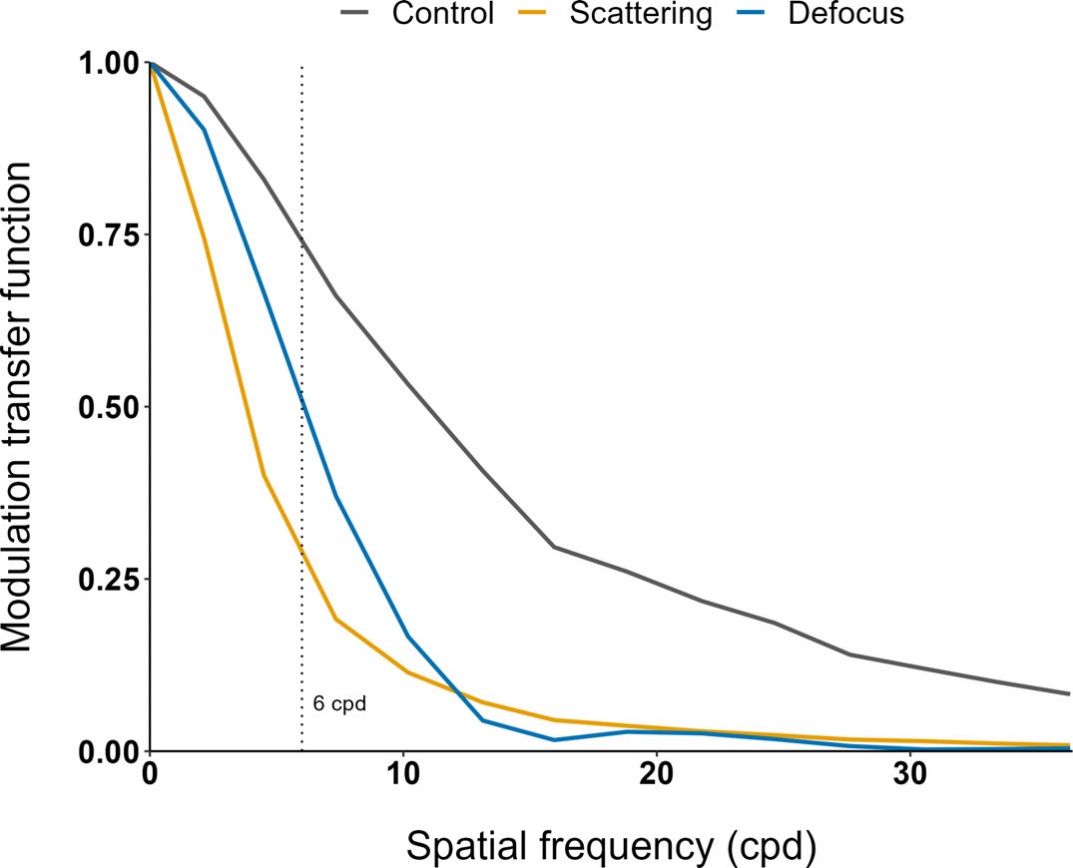
MTF in the range of spatial frequencies from 0 to 36 cpd for the different adaptation lens conditions (control, scattering, and defocus) for the average human eye (Watson, 2013). The dashed line indicates the tested spatial frequency of 6 cpd.

The following lens conditions were applied binocularly to degrade the retinal image: Bangerter foil with a density of 0.8 (Breitfeld & Schliekert, Karben, Germany), referred to as “scattering,” and a spherical defocus of +0.5 D, referred to as “defocus.” The dioptric power of defocus was matched to the scattering condition by a visual acuity reduction of one line on the letter vision chart (+0.1 logMAR), comparable to a recent study investigating DOT lenses (Rappon et al., 2022b). Both image degrading conditions were compared to a clear lens control condition. For a more detailed comparison, the lens conditions were optically characterized by a novel system based on the technology of spatial light modulation (Arias et al., 2023). The system had a 532-nm-wavelength laser light source and captured images of the respective point spread function under central illumination. The modulation transfer function (MTF) was given by the absolute value of the Fourier transform of the point spread function. Subsequently, the MTF data for the lens was multiplied pointwise by the averaged human MTF values for a pupil diameter of 5 mm (Watson, 2013). The MTF results for the lens conditions in the human eye are presented in Figure 2.

### Study procedure

The effect of each condition on neural contrast sensitivity was investigated on separate days at the same time of the day to avoid diurnal fluctuations in vision or fatigue (Foster & Hankins, 2007; Tassi & Pins, 1997). To account for daily variabilities in visual performance, for each lens condition a baseline was recorded before adaptation, which was then compared to the measurements after exposure to this lens. The conditions were randomized to minimize any training effects due to the unusual stimulus presentation method. The respective test lenses were mounted in the aforementioned trial frame in addition to the individual's spherocylindrical correction. The study procedure involved a 10-minute rest adaptation, where the participants sat relaxed in a mesopic lighted room without any visual or physical task. The relaxation phase was followed by a neural contrast sensitivity test training round to ensure equal starting conditions for the primary investigation among the participants. Following this, the baseline was determined by pooling across six pre-adaption neural contrast sensitivity measurements. Subsequently, the participants underwent a 30-minute adaptation to the respective lens condition. During the adaptation phase, the participants watched a movie that mimicked indoor scene viewing in a room with 350-lux lighting from a distance of 5 meters with a field of view of 3.66° to avoid accommodation. The mean pupil diameter of the participants under these conditions was similar across the lens conditions, measuring 4 mm on average when assessed prior to adaptation. Finally, six post-adaptation neural contrast sensitivity measurements were performed to determine possible changes as a result of the adaptation to the lens condition. The neural contrast adaptation reported in the following was defined by the difference in neural contrast sensitivity between the baseline and the respective post-adaption dataset. Neural contrast sensitivity measurements were taken on the dominant eye of each participant without any lens in front of the eye. The dominant eye was determined by having each participant look through the small aperture between their interlaced fingers. The visible eye from the examiner's side through the aperture constituted their dominant eye, following a standard clinical procedure (Crider, 1944; Fink, 1938; Mapp, Ono, & Barbeito, 2003; Porac & Coren, 1976).

### Statistical analysis

Data were analyzed using R (R Core Team, 2020). Participant information regarding refractive error and axial length is described as mean and standard deviation (*SD*). Neural contrast sensitivity and neural contrast adaptation (determined by changes in post-adaptation neural contrast sensitivity from pre-adaptation neural contrast sensitivity) are reported as mean and standard error (*SE*) in logarithmic scale units (log contrast sensitivity [logCS]). Before statistical analysis, datasets underwent outlier removal using a boxplot analysis, with data points < 1.80 logCS and > 2.55 logCS excluded. The datasets were then grouped separately for the performed measurement number (1–6), time (pre- or post-adaptation), and condition (control, scattering, or defocus) across the participants. Pre-adaptation data points 1.5 interquartile ranges (IQRs) outside of the median were removed. To allow for differences in neural contrast sensitivity after adaptation to the lens condition, a larger outlier range was selected for the post-adaptation results. Therefore, data points 3 IQRs away from the median were excluded from further analysis. Furthermore, pre-adaptation data points were averaged across the six consecutively performed contrast sensitivity measurements (per condition and for each participant). Post-adaptation data points were grouped for the first three (1–3) and the second three (4–6) measurements. The change of post-adaptation data (for each measurement group) was calculated from the averaged pre-adaptation neural contrast sensitivity. The repeatability, the 95% repeatability limit (McAlinden, Khadka, & Pesudovs, 2011), and the time efficiency of the testing procedure were analyzed based on the neural contrast sensitivity results prior to adaptation. Furthermore, the reproducibility was assessed by Bland–Altman analysis (Euser, Dekker, & le Cessie, 2008) and as the intraclass correlation coefficient (ICC) based on the pre-adaptation neural contrast sensitivity values across the three lens conditions. The ICC is interpreted as excellent between 0.75 and 1.00, as good between 0.60 and 0.74, as fair between 0.40 and 0.59, and as poor for values smaller than 0.40 (Cicchetti, 1994). Normal distribution of the data was verified with the Lilliefors test. Statistical analysis was then conducted using repeated-measures analysis of variance (ANOVA) with sphericity correction by Mauchly's sphericity test, as well as post hoc testing of pairwise *t*-tests with Bonferroni correction and pairwise Friedman rank-sum test. The following factors were analyzed according to neural contrast sensitivity and adaptation: three levels of condition (control, scattering, and defocus) and two levels of time (pre- and post-adaptation). Additionally, adaptation in dependence on testing time was analyzed by comparing the post-adaptation neural contrast sensitivity from baseline. Because the average trial duration was about 25 seconds (Figure 5), the data were clustered into 25 ± 12.5-second sequences as follows: ≤12.5 seconds, >12.5 to ≤37.5 seconds (referred to as “25 seconds”), >37.5 to ≤62.5 seconds (referred to as “50 seconds”), and so on through >150 seconds (referred to as “>150 seconds”). The clustered data points were analyzed using a linear mixed model and the post hoc test of estimated marginal means, with the dependent variable of neural contrast adaptation, the random effect of participants, and two fixed effects of condition (control, scattering, and defocus) and time (25, 50, 75, 100, 125, 150, and >150 seconds). To note, data for ≤12.5 seconds were removed due to too few data points. The significance level set for all types of analysis was α = 0.05 and significant effects were defined by *p* < 0.05.

## Results

### Participant data

A total of 29 participants were included in the study (22 females) with a mean age of 26 ± 5 years (range, 18–35). The subjective refraction of the dominant eye revealed a spherical equivalent of −1.00 ± 1.82 D (range, −5.25 to 3.88). Biometrical measurements revealed an axial length of 23.90 ± 0.91 mm for the dominant eye.

### Effects on neural contrast sensitivity

**Figure 3.**
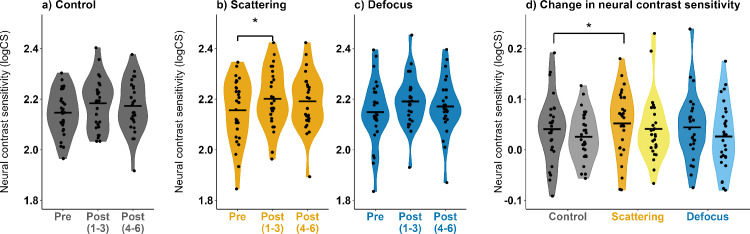
Mean pre- and post-adaptation neural contrast sensitivity values (in logCS; black crossbar) and their distribution for individual lens conditions: (**a**) control, (**b**) scattering, and (**c**) defocus. (**d**) Changes in neural contrast sensitivity to the conditions, averaged for the initial three and last three measurements after removal of the lens condition (*n* = 29 participants).

Neural contrast sensitivity results are presented in Table 1 and Figure 3. Pre-adaptation neural contrast sensitivity was similar among all lens conditions with 2.16 ± 0.02 logCS. A significant increase in neural contrast sensitivity was found in the first three measurements compared with baseline neural contrast sensitivity after exposure to the scattering condition (2.20 ± 0.02 logCS; *F* = 3.26, *p* = 0.04, ANOVA) but not for control (2.18 ± 0.02 logCS) or defocus (2.19 ± 0.02 logCS; both *p* > 0.05, ANOVA). The largest difference was found after adaptation to scattering (+0.05 ± 0.02 logCS), followed by defocus (+0.03 ± 0.02 logCS), and finally after adaptation to control (+0.02 ± 0.02 logCS). A comparison of the change in neural contrast sensitivity across the lens conditions revealed a significant sensitivity increase in the scattering condition compared with the control (*F* = 4.17, *p* = 0.04, Friedman test) for the first three measurements. No effects were found between scattering and defocus or defocus and control (all *p* > 0.05, Friedman test). For the last three post-adaptation measurements, neural contrast sensitivity was less than for the first three post-adaptation measurements (2.19 ± 0.02 logCS for both control and scattering; 2.18 ± 0.02 logCS for defocus), and no longer significantly increased compared with baseline (*F* = 0.62, *p* = 0.61, ANOVA). The differences in the observed changes for the last three post-adaptation measurements across the lens conditions were not significant (+0.04 ± 0.01 logCS, +0.03 ± 0.01 logCS, and +0.03 ± 0.01 logCS, for scatter, control, and defocus, respectively; *F* = 0.42, *p* = 0.66, ANOVA).

**Table 1.** Pre-adaptation (averaged across all six measurements) and post-adaptation (averaged across the first three measurements and the second three measurements) neural contrast sensitivity and the differences (mean ± *SE*) in logCS for the control, scattering, and defocus conditions (*n* = 29 participants).

Measurement	Control	Scattering	Defocus
Pre-adaptation	2.16 ± 0.02	2.16 ± 0.02	2.16 ± 0.02
Post-adaptation (measurements 1–3)	2.18 ± 0.02	2.20 ± 0.02	2.19 ± 0.02
Change, pre- vs. post-adaptation (measurements 1–3)	+0.02 ± 0.02	+0.05 ± 0.02	+0.03 ± 0.02
Post-adaptation (measurements 4–6)	2.19 ± 0.02	2.19 ± 0.02	2.18 ± 0.02
Change, pre- vs. post-adaptation (measurements 4–6)	+0.03 ± 0.01	+0.04 ± 0.01	+0.03 ± 0.01

**Figure 4.**
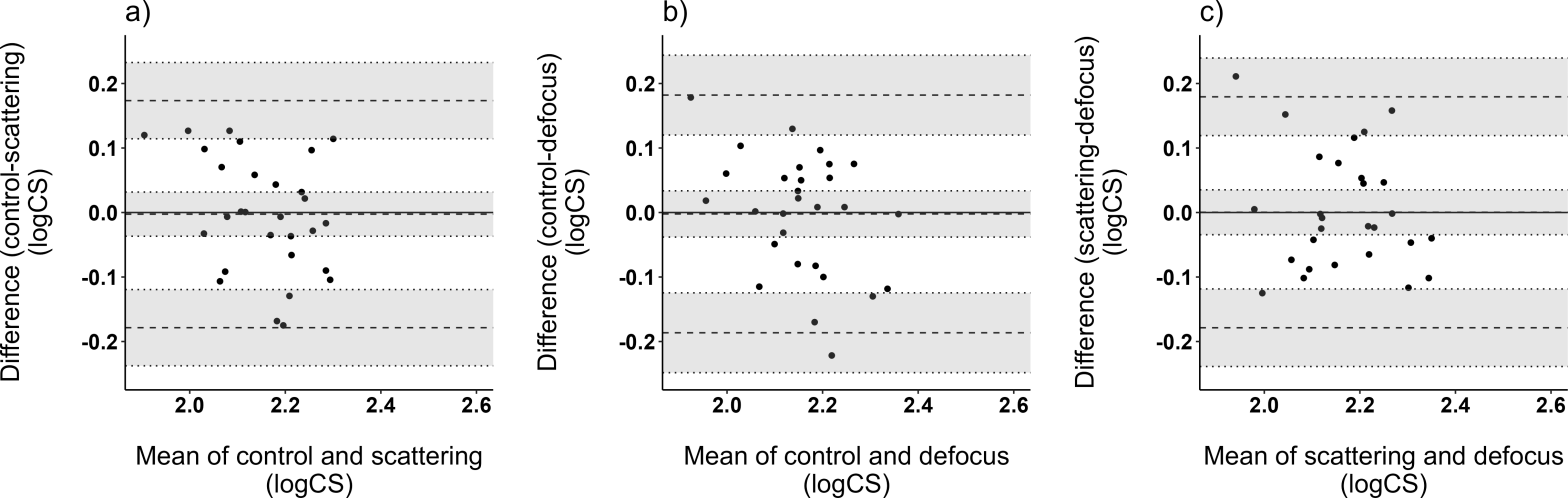
Bland–Altman plots of pre-adaptation neural contrast sensitivity (in logCS) of the lens conditions (**a**) control versus scattering and (**b**) control versus defocus and (**c**) scattering versus defocus, showing the mean differences and 95% confidene intervals as dashed lines (*n* = 29 participants).

Analysis of pre-adaptation neural contrast sensitivity measurement repetitions showed repeatability for the method of adjustment procedure of ±0.01 logCS (95% repeatability limit of ±0.03 logCS). The reproducibility calculated by the ICC between averaged pre-adaptation measurements of the lens conditions resulted in 0.87, with a mean difference of −0.008 logCS (95% confidence interval, −0.22 to 0.21). Additionally, the difference in pre-adaptation neural contrast sensitivity between the lens conditions was compared by Bland–Altman analysis. A mean difference of −0.002 logCS was found between control and scattering (95% limits of agreement [LoA], −0.18 to 0.17) (Figure 4a). The comparison of control and defocus revealed a mean difference in pre-adaptation contrast sensitivity of −0.002 logCS (95% LoA, −0.19 to 0.18) (Figure 4b). Figure 4c shows the agreement of pre-adaptation neural contrast sensitivity between scattering and defocus with a mean difference of 0.0003 logCS (95% LoA, −0.18 to 0.18). The results show excellent agreement among the lens conditions.

### Time course of adaptation

**Figure 5.**
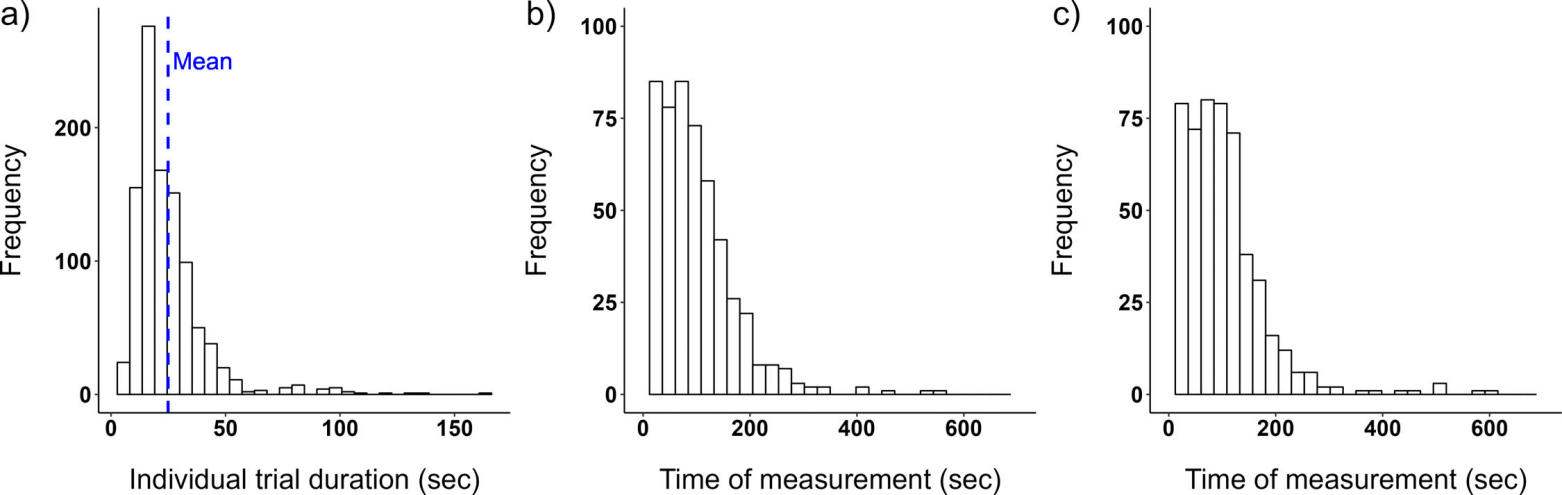
(**a**) The distribution of the individual adjustment trial duration (in seconds). (**b**, **c**) The response time (in seconds) across the six performed measurements before adaptation to a lens condition (**b**) and after adaptation to the lens condition (**c**) (*n* = 29 participants).

The individual trial duration was on average 25 ± 16 seconds (minimum, 6 seconds; maximum, 164 seconds) (Figure 5a). The response time across the six performed measurements before adaptation to the lens condition is presented in Figure 5b and after adaptation in Figure 5c. The first three post-adaptation measurements were finished within 88 ± 53 seconds, and the second three at 171 ± 82 seconds.

**Figure 6.**
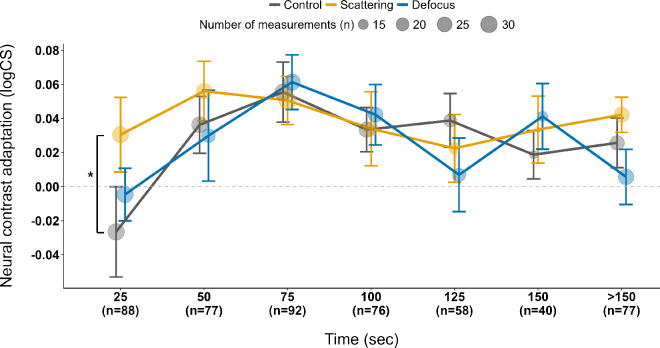
Change in neural contrast sensitivity of pre- versus post-adaptation (mean ± *SE*) grouped by the time of the performed measurement with a range of ±12.5 seconds for the adaptation lens conditions of control, scattering, and defocus (*n* = 29 participants).

Neural contrast sensitivity alterations in dependence on the testing time are displayed in Figure 6. Adaptation to the scattering condition shows quantitatively the greatest improvement in neural contrast sensitivity across the testing time when compared to the control and defocus conditions, except between 75 ± 12.5 seconds and 150 ± 12.5 seconds of measurement time. At 25 ± 12.5 seconds, the change in neural contrast sensitivity from baseline observed for scattering was significantly higher compared with the control (+0.03 ± 0.03 logCS vs. −0.03 ± 0.02 logCS; *F* = 4.17, *p* = 0.04, Friedman test). At all other time sequences, no statistically relevant effect was found among the lens conditions (all *p* > 0.05).

### Refractive status as a covariate

**Figure 7.**
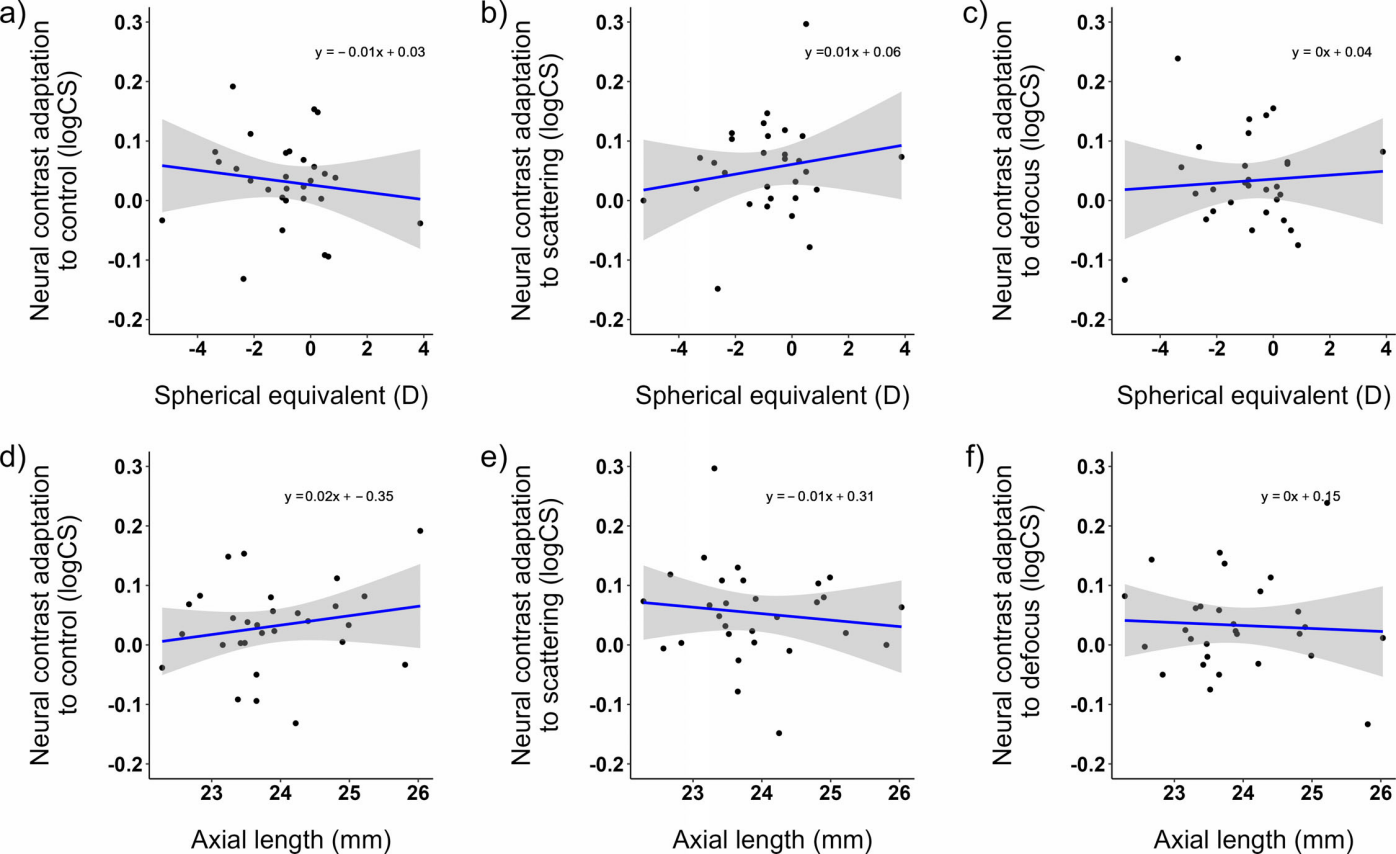
(**a**–**c**) Correlation of neural contrast adaptation to the conditions of control (**a**), scattering (**b**), and defocus (**c**) for the initial three measurements (in logCS) and spherical equivalent refractive error (in diopters). (**d**–**f**) Correlation of neural contrast adaptation to the conditions of control (**d**), scattering (**e**), and defocus (**f**) for the initial three measurements and axial length (*n* = 29 participants). Gray shaded areas show the 95% confidence intervals for the linear correlations.

To test, if the refractive status of the eye influences adaptation, the measured neural contrast adaptations for the control, scattering, and defocus conditions were correlated with the individual spherical equivalent refractive errors (Figures 7a to 7c) and axial length (Figures 7d to 7f). No significant correlation was found across the conditions (all *p* > 0.05).

## Discussion

This study investigated changes in neural contrast sensitivity after short-term adaptation to scattering and defocus. A significant change of neural contrast sensitivity from baseline was found after scatter adaptation in the first three post-adaptation measurements, as it was significantly increased compared to the control condition. The adaptation effect did not last over the last three post-adaptation measurements. Neural contrast sensitivity changes were not significant for the control and defocus lens conditions. Neural contrast sensitivity depending on the measurement time revealed a significant sensitivity increase after adaptation to scattering compared with the control at 25 ± 12.5 seconds. The observed gains in neural contrast sensitivity tend to be quantitatively highest for scatter; however, these results indicate that the after-effects do not last long.

### Neural contrast sensitivity effects

The size of the effect of scattering (+0.05 logCS) was marginally larger than the step size of the neural contrast sensitivity test, based on the condition-related pre-adaptation neural contrast sensitivity; however, the adaptation effect to scattering was significantly different from the control condition. With the exhibited effect size and given the number of participants (29), a power of 84% was achieved. Pre-adaptation neural contrast sensitivity showed similar means and variations among the conditions, as shown in the Bland–Altman plots (Figure 4). Thus, a bias between the exhibited effects related to the pre-adaptation measurement can be excluded and the found changes were persistent independent of the separate measurement days.

The observed absolute, non-significant changes in relation to the control condition might be explained best by a perceptual learning effect, which was also evident in previous studies (Sagi, 2011). Notably, the change in neural contrast sensitivity following adaptation to scattering was even greater and significantly increased compared with the control condition, indicating a meaningful effect and ruling out a pure learning effect. Although neural contrast adaptation to scattering has not been investigated so far, previous studies have found contradictory contrast sensitivity results regarding adaptation to optically induced scattering using Bangerter foils. Including a clear central zone in the middle of the lens, no central contrast adaptation to a Bangerter foil of density 0.8 was found in another study (Neumann et al., 2022). Villa-Carpes et al. (2021) found an increase in visual acuity after a 40-minute adaptation period to Bangerter foils with a density of 0.6. In other studies measuring regular contrast sensitivity directly after the application of Bangerter foils (without any adaptation phase to the foils), a direct decrease was found in contrast sensitivity over a spatial frequency range of 1.5 to 12 cpd (Breher et al., 2023; Neumann, Breher, & Wahl, 2021; Pérez et al., 2010; Perez, Manzanera, & Artal, 2009) and in visual acuity (Pérez et al., 2010; Villa-Carpes et al., 2021). Furthermore, the current study found no neural adaptation to induced defocus. Adaptation to defocus has extensively been studied in the past, with reports of increases in contrast sensitivity in the mid-spatial frequency range (Venkataraman, Winter, Unsbo, & Lundström, 2015), such as at 3.22 cpd after a 10-minute adaptation to +4 D defocus (Ohlendorf & Schaeffel, 2009) and at 8 cpd and 12 cpd after a 30-minute adaptation to +2 D defocus (Rajeev & Metha, 2010). In this study, a spherical defocus of +0.5 D was applied to achieve the same visual acuity reduction as the applied scattering condition, near a depth of focus of 0.5 D (Vinas, Dorronsoro, Cortes, Pascual, & Marcos, 2015). In comparison with defocus-based optical myopia control strategies, the defocus lens applied in the current study was low in dioptric power. However, it must be taken into account that myopia control strategies are currently applied in the periphery, whereas this study aimed to investigate effects within the fovea. To translate peripheral lens characteristics into requirements for a central lens design having a similar impact on the perceived image, the structure of retinal receptive fields must be considered. At an eccentricity of 8° (roughly the onset of the blurred region for a lens with a 9-mm clear zone), the midget ganglion cell receptive field size is 4.4 arcmin (Watson, 2014). Peripheral-induced defocus lenses, such as the DIMS lenses, with a defocus power of +3.5 D generate a point spread function with a full width at half maximum (FWHM) of 67 arcmin. Thus, the point spread function of the DIMS corresponds to the 17-fold receptive field size. In the foveola, the receptive field size of midget ganglion cells corresponds directly to cone diameter (0.5 arcmin) due to a 1:1 connectivity. If the blurred point spread function should spread comparably across the ganglion cells in the fovea, the FWHM of the point spread function must be 8.5 arcmin. This requirement is quite accurately met when using a defocus of +0.5 D (FWHM = 8.6 arcmin). A low impact of the applied lens conditions on visual acuity is relevant, as visual acuity is an important parameter not only with regard to the quality of life (Brown, 1999) but also for school-aged children in education and normal ocular development (Ikeda, 1980).

The absence of an adaptation effect for spherical defocus can be explained by the lower impact on image contrast when compared to scattering. Considering the whole range of spatial frequencies, contrast is reduced differently by scattering and defocus between 0 and 20 cpd, before it aligns for spatial frequencies greater than 20 cpd. At the tested spatial frequency of 6 cpd, optical scattering reduced the MTF amplitude by approximately half compared with defocus (MTF_scattering_ = 0.29 vs. MTF_defocus_ = 0.51) (Figure 2). Furthermore, it is worth noting that a slight contrast modulation was also present in the clear control condition at 6 cpd compared with an aberration-free environment, which is negligible compared to the scattering and defocus conditions. Another possible explanation of the observed adaptation effects is a distinct retinal and neural processing of scattering and defocus. However, this could not be addressed within the current study and requires further experiments. In previous investigations, optical characterization of scattering and defocus lenses revealed differences in retinal image contrast (Arias et al., 2023; Pérez et al., 2010). Here, scattering led to a reduction in retinal image contrast and defocus to retinal image sharpening and contrast increase (Arias et al., 2023).

In this study, the participants received input from a wide range of spatial frequencies during the 30 minutes of adaptation. This approach has been used in past studies (Ohlendorf & Schaeffel, 2009; Ohlendorf, Tabernero, & Schaeffel, 2011; Venkataraman et al., 2015). According to previous literature, indoor scenes, including movies played on a display, range from 0 to 100 cpd (Bex, Solomon, & Dakin, 2009; Flitcroft et al., 2019). However, no detailed frame-by-frame analysis of the video material has been carried out, and no over- or under-representation of certain spatial frequencies or orientations has been determined. The contrast reduction caused by the adaptation conditions in the current study affected the spatial frequency channels differently over the entire range. The found adaptation effect to scattering at 6 cpd is best explained by the amount of contrast reduction over the range of spatial frequencies. An additional explanation for these adaptation effects to scattering and defocus is a required minimum amount of contrast reduction to adjust the contrast gain of underlying neural mechanisms (DeAngelis, Ohzawa, & Freeman, 1995). With adjustment of the contrast gain, the best possible image is perceived. In the present study, this enhancement of neural contrast sensitivity persisted shortly after the lenses were removed. These aftereffects were also found in previous studies (Magnussen & Greenlee, 1985; Ohlendorf & Schaeffel, 2009). The human eye is sensitive to lower spatial frequencies of natural scenery, as low or medium spatial frequencies up to 6 cpd are necessary to identify natural scenes (Bex et al., 2009). In addition, low spatial frequencies are more rapidly processed in the cortex compared with higher spatial frequencies (Kauffmann, Chauvin, Guyader, & Peyrin, 2015). Therefore, this study was performed at a spatial frequency of 6 cpd, the peak sensitivity of the contrast sensitivity function (Blakemore & Campbell, 1969a; Michael et al., 2011), where any potential changes would be mostly visible (Campbell & Green, 1965). Furthermore, neural contrast sensitivity of only one spatial frequency was tested due to time restraints regarding the relatively short aftereffects (Greenlee, Georgeson, Magnussen, & Harris, 1991; Ohlendorf & Schaeffel, 2009).

### Time course of contrast adaptation

This study investigated short-term adaptation after 30 minutes. Adaptation periods in past studies range from minutes to weeks (Khan, Dawson, Mankowska, Cufflin, & Mallen, 2013; Rutstein et al., 2011; Villa-Carpes et al., 2021). Contrast adaptation to scattering has been found after 40 minutes (Villa-Carpes et al., 2021) and to defocus already after 4 minutes (Khan et al., 2013). One study that investigated visual acuity after 6 and 12 weeks of wearing Bangerter foils during the daytime reported a significant increase in visual acuity (Rutstein et al., 2011). Further research is required on beneficial time periods to enhance visual performance and possibly beneficial effects on structural changes. In the current study, a significant adaptive aftereffect compared to control was found for scattering at 25 ± 12.5 seconds. A negative, but not significant, aftereffect was exhibited for the control condition at this specific time, as neural contrast sensitivity increased after 25 ± 12.5 seconds. The negative shift in the control condition could be explained by the adaptation process itself (Blakemore & Campbell, 1969a), despite the flickering stimulus to minimize adaptation effects. This difference in the scatter to the control condition did not last long, which is consistent with previous investigations, which have reported aftereffects of short duration (2–5 minutes) (Greenlee et al., 1991; Magnussen & Greenlee, 1985; Ohlendorf & Schaeffel, 2009).

### Contrast adaptation in the context of emmetropization

Contrast adaptation and processing are assumed to play a role in myopia development based on eye growth (Aleman, Wang, & Schaeffel, 2018; Diether et al., 2001; Ohlendorf & Schaeffel, 2009; Smith & Hung, 2000; Stoimenova, 2007). Adaptation to induced myopic defocus seems to benefit myopia control by reducing axial length progression and increasing choroidal thickness, accompanied by an improvement in contrast sensitivity, whereas imposed hyperopic defocus causes the opposite (Jonas, Ohno-Matsui, & Panda-Jonas, 2019; Ohlendorf & Schaeffel, 2009). Interestingly, adapting to a low induced defocus of +0.5 D, also known as under-correction of myopia, has shown absolute myopia progression, albeit not statistically significant myopia (Adler & Millodot, 2006; Chung, Mohidin, & O'Leary, 2002; Lin et al., 2015; Vasudevan, Esposito, Peterson, Coronado, & Ciuffreda, 2014). Literature is inconclusive with respect to the effect of scattering on myopia control. The use of scattering-inducing Bangerter foils is also prevalent in animal studies and has led to form-deprivation myopia (Smith & Hung, 2000). However, in a current human clinical trial, scattering lenses resulted in a slowing of myopia progression (Neitz, Wagner-Schuman, Rowlan, Kuchenbecker, & Neitz, 2022). In a clinical study, a novel myopia control lens was used based on the hypothesis that abnormal high contrast signaling, caused by genetic cone mutations, would lead to myopia (Hagen et al., 2019). Here, it has been suggested that the reduction of retinal image contrast rebalances ON and OFF receptive field processing of the retinal ganglion cells, which controls myopia development. Asymmetric receptive field processing was not demonstrated in a previous study (Breher et al., 2023) and was not investigated in the current study.

Further to this theory, a difference between emmetropic and myopic neural contrast adaptation would be expected; however, in the current study, no significant correlation was found between neural contrast adaptation and refractive error or axial length. Consequently, the refractive status did not influence the adaptation effect. This might be explained by the level of ametropia. In addition to one highly myopic and one hyperopic participant, only emmetropic or low myopic participants took part in this study. To investigate neural adaptation effects in more detail, more high myopic participants should be included in future studies. Furthermore, only the combined retinal and cortical response was measured within the psychophysical approach of the current study. Previous studies have reported the sole responsibility of the retina for emmetropization (Aleman et al., 2018; Carrillo-Aleman, Wang, & Schaeffel, 2019; Hoseini-Yazdi, Read, Alonso-Caneiro, & Collins, 2021; Swiatczak & Schaeffel, 2022; Wallman & Winawer, 2004; Wang, Aleman, & Schaeffel, 2019). Even though it was not possible to trace the adaptation effect back to the retina, it is essential to understand that such an effect exists.

### Neural contrast sensitivity assessment

The baseline (pre-adaptation) results found in the current study are comparable with previous measurements of the neural contrast sensitivity (Campbell & Green, 1965; Suchkov et al., 2021). In addition to these qualitative results, repeatability was also tested in the current study, resulting in a repeatability of ±0.01 logCS (95% repeatability limit of ±0.03 logCS). Previous studies showed poorer repeatability ranging from 0.15 to 0.40 logCS at 6 cpd for regular contrast sensitivity forced-choice procedures (Schilling, Ohlendorf, Leube, & Wahl, 2017). Another type of adjustment contrast sensitivity test reported a coefficient of repeatability of 0.13 logCS at 6 cpd (Neumann et al., 2021). The neural contrast sensitivity results measured by the interferometric system showed excellent reproducibility with an ICC of 0.87. The measurements included in the reproducibility evaluation were performed on different days, so natural daily fluctuation in visual performance could have occurred (Tassi, Pellerin, Moessinger, Hoeft, & Muzet, 2000; Tassi & Pins, 1997), which may not result in an ICC of 1.00. This is in alignment with previous test procedures showing good and excellent reproducibility (Schilling et al., 2017). Hence, the test procedure for the current study can be considered highly repeatable and having excellent reproducibility. The adjustment test method in this study showed a time duration of 25 ± 16 seconds per single run, which is similar to a regular adjustment contrast test reporting 18 seconds (Neumann et al., 2021). Forced-choice procedures have resulted in longer duration times, ranging from approximately 1 to 2 minutes (Schilling et al., 2017). Time efficiency was a goal in the current study, so that fast (de-)adaptation effects could be observed. Further, it is known that long testing procedures lower the attention of the participant (Dietze, 2008; Schilling et al., 2017). According to the current study results, the method of adjustment, newly introduced as a psychophysical procedure in combination with the system, has the potential to be a repeatable and fast test procedure, despite being time unlimited per se (Pelli & Bex, 2013; Pelli & Farell, 1995; Wier, Jesteadt, & Green, 1976). It is noteworthy that the method of adjustment involves an individual criterion to set the contrast threshold (Gescheider, 2013); therefore, the results might be systematically affected by the individually defined criterion from each participant (Lu & Dosher, 2013). To mitigate the potential bias, all participants were required to undergo all test conditions, and changes in neural contrast sensitivity were calculated individually. To monitor an eventual bias, the adaptation period to a clear control condition was implemented, as well as baseline measurements before adaptation. Analysis of pre-adaptation neural contrast sensitivity across all conditions (see Bland–Altman plots in Figure 4) revealed that the pre-adaptation neural contrast sensitivity was stable for each participant, and the changes in neural contrast sensitivity can be assigned to post-adaptation neural contrast sensitivity. In summary, the analysis of repeatability, reproducibility, and agreement showed that the individual criterion was maintained across the study. Furthermore, the interferometric system eliminated optical aberrations of each participant and therefore ruled out the possibility of any impact on neural contrast sensitivity, which is an important advantage of the test procedure.

The method of adjustment has been used before for measurements of regular contrast sensitivity (Burr & Ross, 1982; Neumann et al., 2021; Norton, Corlis, & Bailey, 2002; Wier et al., 1976). It is one of the simplest psychophysical procedures for threshold estimation; however, due to adaptation during the adjustment of the stimulus (Werner, Sharpe, & Zrenner, 2000), thresholds tend to be higher compared with forced-choice procedures (Wier et al., 1976). To avoid this limitation, the stimulus had a 1-Hz flicker. The measurements were also averaged, following recommendations from previous literature suggesting averaging when using the method of adjustment (Lu & Dosher, 2013; Pelli & Bex, 2013; Pelli & Farell, 1995). It has been suggested that the interference technique used in this study is more direct than calculation of the neural transfer function out of the contrast sensitivity function and the modulation transfer function (Leube et al., 2018). Another advantage of assessing the neural contrast sensitivity in comparison with the regular contrast sensitivity is that the visual performance is not limited by the optics of the eye, especially higher order aberrations (Ghosh et al., 2017). Furthermore, neural contrast sensitivity is not affected by pupil size, as optical aberrations are eliminated by the measurement procedure (Campbell & Green, 1965; Dressler & Rassow, 1981; Suchkov et al., 2021). This differs from the regular contrast sensitivity metric, as the pupil here influences the optics and thus aberrations of the eye (Michael et al., 2011; Sanz Diez, Wahl, Schaeffel, & Ohlendorf, 2019; Strang, Atchison, & Woods, 1999). Furthermore, clinical measurements of the neural transfer function have already been used for perceptual training (Artal et al., 2004; Sabesan, Barbot, & Yoon, 2017; Vohnsen, 2021). Direct measurement of neural processes might be also useful not only in understanding physiological visual–neural processes but also in the detection of neural disorders. However, the amount of contrast reduction, illuminance, and intraocular scattering on the retinal level is dependent on pupil size (Arias et al., 2023; Castro-Torres, Martino, Casares-López, Ortiz-Peregrina, & Ortiz, 2021; Vohnsen, 2021), which was measured in the current study before adaptation but not controlled during the adaptation period. Similar pupil sizes can be assumed, as the participants were of similar age and ethnicity (Guillon et al., 2016; Heine, Yazdani, & Wilhelm, 2013).

Additionally, the same room lighting was ensured for all participants during the adaptation period. Effects of pupil diameter on neural contrast sensitivity itself were likely negligible, as the optical aberrations were bypassed (Watson & Yellott, 2012). The lack of a strong effect rather than just a tendency after adaptation to optical scattering might have been caused by the relatively short adaptation period. Thus, long-term effects in the range of weeks to years cannot be explained with it. By using the same Bangerter foil among the participants, differences in manufacturing of the foils and thus differences in contrast reduction levels were controlled for (Perez et al., 2009). Moreover, a Bangerter foil with microbubble patterns aligns with the novel myopia lens featuring microdiffusers. Although their manufacturing methods differ, both aim to scatter light, thus reducing visual acuity and contrast. Furthermore, this study investigated the effect of contrast reduction at the peak of the contrast sensitivity function of 6 cpd (Blakemore & Campbell, 1969a; Michael et al., 2011). The impact on lower or higher spatial frequencies was not examined. Also, higher contrast reductions and long-term effects were not included in the current study.

In summary, the alteration of neural contrast sensitivity at peak sensitivity after adaptation to optically induced scattering might be an underlying mechanism for ocular growth regulation. The question remains, then, how the mechanism controls eye growth.

## Conclusions

Short-term adaptation to reduced retinal image contrast induced by scattering and defocus was psychophysically assessed at the contrast sensitivity function peak in young adults. A significantly higher increase in neural contrast sensitivity was found after adaptation to scattering in the first three measurements after lens removal compared with the control condition. Observed increases in neural contrast sensitivity from baseline after exposure to defocus and control were not significant, most likely related to the impact on contrast reduction at the tested spatial frequency of 6 cpd. Furthermore, neither axial length nor spherical equivalent was correlated with the observed alteration in neural contrast sensitivity after adaptation in any of the lens conditions and therefore did not influence the adaptation effect. Our findings indicate that scattering affected neural contrast sensitivity at peak sensitivity more than in the control and defocus conditions and therefore might be suitable as a myopia control strategy. Further research is required to determine the impact of optically induced scattering and defocus on a wide range of spatial frequencies and to define the minimal contrast reduction necessary to induce a beneficial effect in ocular growth regulation.
